# Gastroesophageal Reflux Disease and Atrial Fibrillation: A Nationwide Population-Based Study

**DOI:** 10.1371/journal.pone.0047575

**Published:** 2012-10-15

**Authors:** Chin-Chou Huang, Wan-Leong Chan, Jiing-Chyuan Luo, Yu-Chun Chen, Tzeng-Ji Chen, Chia-Min Chung, Po-Hsun Huang, Shing-Jong Lin, Jaw-Wen Chen, Hsin-Bang Leu

**Affiliations:** 1 Department of Medical Research and Education, Taipei Veterans General Hospital, Taipei, Taiwan, Republic of China; 2 Division of Cardiology, Department of Medicine, Taipei Veterans General Hospital, Taipei, Taiwan, Republic of China; 3 Healthcare and Management Center, Taipei Veterans General Hospital, Taipei, Taiwan, Republic of China; 4 Division of Gastroenterology, Department of Medicine, Taipei Veterans General Hospital, Taipei, Taiwan, Republic of China; 5 Department of Family Medicine, Taipei Veterans General Hospital, Taipei, Taiwan, Republic of China; 6 Cardiovascular Research Center, National Yang-Ming University, Taipei, Taiwan, Republic of China; 7 Institute of Pharmacology, National Yang-Ming University, Taipei, Taiwan, Republic of China; 8 Institute of Clinical Medicine, National Yang-Ming University, Taipei, Taiwan, Republic of China; 9 Institute of Hospital and Health Care Administration, National Yang-Ming University, Taipei, Taiwan, Republic of China; 10 Faculty of Medicine, National Yang-Ming University, Taipei, Taiwan, Republic of China; 11 Department of Medical Research and Education, National Yang-Ming University Hospital, Yi-Lan, Taiwan, Republic of China; 12 Institute of Biomedical Sciences, Academia Sinica, Taipei, Taiwan, Republic of China; Medical University Innsbruck, Austria

## Abstract

**Objectives:**

Precise mechanisms of atrial fibrillation (AF) are uncertain, but their association with esophageal disorders has been recently proposed. The association between gastroesophageal reflux disease (GERD), the most common gastroesophageal disorder, and AF remains undetermined. We therefore aimed to investigate the association between GERD and later development of AF.

**Methods and Results:**

Patients with GERD were identified from the 1,000,000-person cohort dataset sampled from the Taiwan National Health Insurance database. The study cohort comprised 29,688 newly diagnosed adult GERD patients; 29,597 randomly selected age-, gender-, comobidity-matched subjects comprised the comparison cohort. Cox proportional hazard regressions were performed as a means of comparing the AF-free survival rate for the two cohorts. During a maximum three years of follow-up, a total of 351 patients experienced AF, including 184 (0.62%) patients in the GERD cohort and 167 (0.56%) in the control group. The log-rank test showed that patients with GERD had significantly higher incidence of AF than those without GERD (p = 0.024). After Cox proportional hazard regression model analysis, GERD was independently associated with the increased risk of AF (hazard ratio, 1.31; 95% confidence interval, 1.06–1.61, p = 0.013).

**Conclusion:**

GERD was independently associated with an increased risk of future AF in a nationwide population-based cohort.

## Introduction

Atrial fibrillation (AF) is the most common arrhythmia, and its incidence has risen rapidly in countries with rapidly aging populations [1]. AF may cause disability leading to heart failure, stroke, death, and impaired quality of life, highlighting the importance of the understanding of underlying mechanisms and treatment of AF in clinical practice. However, the precise mechanisms that cause AF are not completely understood. Because of frequent concurrence with other cardiovascular diseases, including coronary artery disease, valvular heart disease, hypertension, and congestive heart failure, etiologies such as hemodynamic stress, atrial ischemia, and neurohumoral cascade activation have been proposed as related to AF. Furthermore, factors such as systemic inflammation [2], sleep apnea [3], alcohol use [4], and specific genetic mutations [5]–[7] have also been reported to be associated with the occurrence of AF.

Gastroesophageal reflux disease (GERD) is one of the most common gastroesophageal disorders. Because of the close positioning of the esophagus and the atria and similar nerve innervations, it has been proposed that AF development could be associated with the occurrence of GERD [8]–[13]. However, the association between GERD and AF has been controversial and inconsistent due to limited sample sizes or cross-sectional study designs [14]. We therefore conducted a nationwide population-based study using the Taiwan National Health Insurance database to investigate the relationship between GERD and later development of AF using a prospective, nationwide, and case-cohort study design.

## Materials and Methods

### Database

The National Health Insurance program in Taiwan has operated since 1995 and enrolls nearly all the inhabitants of the country (21,869,478 beneficiaries out of 22,520,776 inhabitants at the end of 2002) [15]. Thus National Health Insurance covers 99.5% of the population in Taiwan, and the insurance claims database is open to applicant use for medical research purposes. It is one the largest nationwide population-based databases in the world. Currently, the National Health Research Institute (NHRI) in Miaoli, Taiwan manages the National Health Insurance Research Database (NHIRD) and has published several dozen extracted datasets for researchers [16], [17]. The NHRI has released a cohort dataset composed of 1,000,000 randomly sampled people who were alive during 2000 and has collected all records on these individuals. These random samples have been confirmed by the NHRI to be representative of the Taiwanese population. In this cohort dataset, each patient's original identification number has been encrypted to protect privacy. But the encrypting procedure is consistent, so that the linkage of claims belonging to the same patient is feasible within the NHIRD. This study was exempt from full review by the Institutional Review Board in Taipei Veterans General Hospital, since the dataset used consisted of de-identified secondary data released to the public for research purposes. All data was analyzed anonymously.

### Study Patients

We identified the patients with GERD from the 1,000,000-person sampled cohort dataset. The following patients were included: (1) patients who were 18 years old or older; (2) patients who were diagnosed with GERD (*International Classification of Diseases, Ninth Revision, Clinical Modification* (ICD-9-CM) codes 530.11 or 530.81). We excluded patients who had been diagnosed with GERD or arrhythmia before enrollment. The Bureau of National Health Insurance requires that GERD patients be diagnosed by either endoscopy or 24-hour pH-meter inspection before proton pump inhibitor (PPI) can be prescribed for treatment. The diagnoses of GERD are valid, and this criteria has been used in similar studies [14], [18]. A control group was selected from patients without a history of esophageal diseases or arrhythmia. Age and co-morbidities were matched in the two groups. Comorbidities included preexisting (in the year before treatment) hypertension, diabetes mellitus, hyperlipidemia, congestive heart failure, coronary artery disease, ischemic stroke, chronic obstructive pulmonary disease, and thyrotoxicosis.

### Atrial Fibrillation Event Measurement

The endpoint of the study was occurrence of administrative claims with AF (ICD-9-CM code 427.31) as the main diagnosis during hospitalization or subsequent outpatient visits. Diagnosis of AF was confirmed by electrocardiography and Holter monitors for insurance claim purposes. The identification of AF by insurance claims data is valid and has been used in previous studies [19], [20].

### Statistical Analysis

Microsoft SQL Server 2005 was used for data management and computing. Statistical analysis was performed with SPSS software (Version 15.0, SPSS Inc., Chicago, Illinois, USA). All data were expressed as the frequency (percentage) or mean±standard deviation. We compared parametric continuous data for the case and control groups using unpaired Student's *t*-test. Categorical data between the two groups were compared with the Chi-square test and Yates' correction or Fisher's exact test, as appropriate. We used Kaplan-Meier analysis for survival analysis, with the significance based on the log-rank test. Survival time was calculated from the date of GERD diagnosis to the date of AF diagnosis. Multiple regression analysis was carried out using Cox proportional hazard regression analysis to evaluate the independent factors determining the occurrence of AF. Statistical significance was inferred at a two-sided *p* value of <0.05.

## Results

A total of 29,688 patients (mean age 50.99±16.61 years) with newly diagnosed GERD were identified from the 1,000,000-person sampled cohort dataset. Another 29,597 control subjects, matched for age and co-morbidities including hypertension, diabetes mellitus, hyperlipidemia, congestive heart failure, coronary artery disease, ischemic stroke, chronic obstructive pulmonary disease, and thyrotoxicosis, except GERD, were enrolled as the control group. The demographic parameters of the study subjects are shown in **Table 1**.

**Table 1:** Demographic data on patients with and without gastroesophageal reflux disease.

Variables	GERD (n = 29,688)		Control (n = 29,597)		*p*-value
Age (years)	50.99±16.61		50.85±16.85		0.311
Male	14,373	(48.41%)	14,333	(48.43%)	0.974
Hypertension	11,366	(38.28%)	11,324	(38.26%)	0.953
Diabetes mellitus	6,979	(23.51%)	6,931	(23.42%)	0.801
Hyperlipidemia	10,180	(34.29%)	10,137	(34.25%)	0.924
Congestive heart failure	2,003	(6.75%)	1,939	(6.55%)	0.347
Coronary artery disease	8,457	(28.49%)	8,402	(28.39%)	0.792
Ischemic stroke	2,489	(8.38%)	2,431	(8.21%)	0.457
Chronic obstructive pulmonary disease	13,287	(44.76%)	13,233	(44.71%)	0.914
Thyrotoxicosis	1,800	(6.06%)	1,745	(5.90%)	0.396

GERD, gastroesophageal reflux disease.

**Figure 1:**
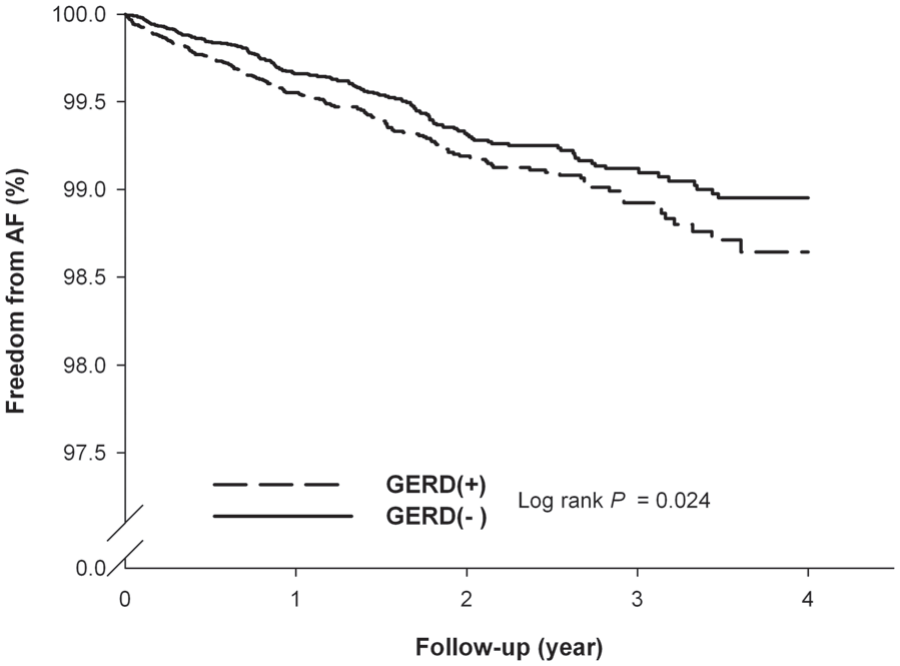
Kaplan-Meier curves of the freedom from atrial fibrillation (AF) in the patients. Group 1 included patients with gastroesophageal reflux disease (GERD), and group 2 included patients without GERD. There was a statistically significant difference between the two curves (log-rank test, *p* = 0.024).

During a maximum three years of follow-up, 184 (0.62%) of the patients with GERD experienced AF occurrence, while 167 subjects (0.56%) from the control group experienced the occurrence of AF. The log-rank test showed that patients with GERD had significantly higher incidence of AF than those without GERD (p = 0.024). **Figure 1** exhibits the results of a Kaplan-Meier analysis. After Cox proportional-hazard model analysis, GERD was independently associated with increased risk of developing AF (hazard ratio [HR], 1.31; 95% confidence interval [CI], 1.06–1.61, p = 0.013) **(** **Table 2** **)**.

**Table 2:** Independent predictors of new-onset atrial fibrillation.

Variable	All patients (n = 59,285)	GERD (n = 29,688)	Control (n = 29,597)
AF			
Yes	351 (0.59%)	184 (0.62%)	167 (0.56%)
No	58,934 (99.41%)	29,504 (99.38%)	29,430 (99.44%)
Crude HR (95% CI)		1.27 (1.03–1.57)	1.00
Adjusted HR (95% CI)		1.31 (1.06–1.61)	1.00

AF, atrial fibrillation; CI, confidence interval; GERD, gastroesophageal reflux disease; HR, hazard ratio.

Adjusted for age, sex, hypertension, diabetes mellitus, hyperlipidemia, congestive heart failure, coronary artery disease, ischemic stroke, chronic obstructive pulmonary disease, and thyrotoxicosis.

We further analyzed our patients according to whether or not PPIs were administered for GERD treatment. There were 12,862 patients who received PPIs and 16,826 patients who did not. GERD patients who received PPIs had increased risk of AF (HR, 1.46; 95% CI, 1.15–1.86, p = 0.002). However, GERD patients who were not prescribed PPIs did not have increased risk of AF (HR, 1.14; 95% CI, 0.86–1.51, p = 0.378) **(** **Table 3** **)**.

**Table 3:** Independent predictors of new-onset atrial fibrillation in patients with gastroesophageal reflux disease according to proton pump inhibitor use.

Variable	Crude HR (95% CI)	*p*-value	Adjusted HR (95% CI)	*p*-value
PPIs				
Yes (n = 12,862)	1.69 (1.33–2.14)	<0.001	1.46 (1.15–1.86)	0.002
No (n = 16,826)	0.91 (0.68–1.20)	0.485	1.14 (0.86–1.51)	0.378

CI, confidence interval; HR, hazard ratio; PPIs, proton pump inhibitors.

Adjusted for age, sex, hypertension, diabetes mellitus, hyperlipidemia, congestive heart failure, coronary artery disease, ischemic stroke, chronic obstructive pulmonary disease, and thyrotoxicosis.

## Discussion

Our current study has demonstrated that GERD was independently associated with an increased risk of AF in a nationwide population-based cohort, suggesting that GERD may play an important role in determining the future risk of developing AF.

GERD, characterized by acid regurgitation or heartburn, is the most common gastrointestinal diagnosis made during visits to outpatient clinics [21]. The annual prevalence rates of GERD in different populations vary between 0.8 and 40% [22]–[26] and continue to grow [26]–[28]. Because of the close positioning of the esophagus and the atria, a possible association between GERD and AF development has been proposed [8]–[13]. However, the association between GERD and AF has not been consistently demonstrated. Kunz et al. [9] first reported that the presence of GERD may increase the risk of AF by 39% (relative risk, 1.39; 95% CI, 1.33–1.45) in a small-scale population, suggesting a close association between AF and GERD. However, Bunch et al. [14] found an inverse relationship between the presence of GERD and AF in a random sample of residents of Olmsted County, Minnesota. Our current study is the first national population-based epidemiological study to investigate the association between GERD and the risk of AF. We found that patients with GERD had significantly higher incidence of AF than those without GERD. Furthermore, GERD was independently associated with increased risk of developing AF.

Although the actual mechanism by which GERD leads to cardiac arrhythmia remains undetermined, certain observational evidence might offer possible explanatory mechanisms. First, GERD could induce vagal nerve stimulation [29], [30]. Accumulating evidence now suggests that the induction of AF may be related to vagal nerve overstimulation [31], [32] and vagal nerve-mediated parasympathetic stimulation [33]. Therefore, vagal nerve overstimulation, which has been observed in patients with GERD, may be responsible for the association between GERD and an increased risk of AF. Second, the precise mechanisms of AF are uncertain, but they have proven to be associated with inflammation [34]. The close anatomical relationship between the esophagus and the atria [35], in addition to the local inflammatory process observed in GERD [36], theoretically provide a mechanism by which GERD initiates AF via the close positioning of the esophagus and the atria. Third, GERD may induce an autoimmune response that contributes to AF [37]. Fourth, acid stimulation of the lower esophagus could cause a significant reduction in coronary blood flow in patients with coronary artery disease [38], and chronic atrial ischemia has been proposed to predispose individuals to AF [39]. Our current study is the first large-scale population-based study that demonstrates the association between GERD and an increased risk of AF. Subsequent in vivo or in vitro studies are needed to provide direct evidence of GERD's role in the pathogenesis of AF.

Several case series have reported a decrease of AF severity after GERD treatment [10]–[14]. Stollberger et al. [11] studied 18 patients with GERD and paroxysmal AF who complained of retrosternal and epigastric pain. After treatment with PPIs, the epigastric pain and inflammation as well as the AF attacks either stopped completely or decreased in frequency. Weigl et al. [12] demonstrated that PPI therapy led to a decrease in AF symptoms in 78% of cases with AF and reflux esophagitis. Furthermore, the decrease was so pronounced that the antiarrhythmic drugs were discontinued in 28% of the patients. Gerson et al. [13] studied three patients with GERD who underwent simultaneous Holter and 24-hour pH monitoring. All of the patients showed a reduction in arrhythmia symptoms with acid suppression therapy. Gillinov et al. [10] reported a case of hiatal hernia. Paroxysmal AF returned to a normal sinus rhythm after GERD symptoms were relieved with a successful Nissen fundoplication. However, the significance of these reports is limited by their small sample sizes. In our current study, we have analyzed our patients according to whether or not PPIs were administered for GERD treatment, and we have found that patients who needed PPIs had a higher risk of developing AF in the future, suggesting that GERD patients with symptoms significant enough to need treatment tend to have higher risk of AF. However, there are some reports that PPIs may be proarrhythmic, which could interfere with this interpretation [40], [41]. The proarrhythmic effect of PPIs remains controversial [10]–[14], [40],[41]. Larger prospective studies or meta-analyses are needed to confirm this hypothesis.

The strength of our study is our use of a population-based dataset, which provides a large-sized sample of subjects and enables us to trace prospectively the association between GERD and AF. However, there are still some limitations in our study. First, the diagnoses of GERD are identified using the ICD-9 codes from the database, and its prevalence may be underestimated because only subjects seeking medical evaluation can be identified by our study. This study was conducted with Taiwan's NHI database, in which the diagnosis was supposed to be confirmed clinically by the individual physicians in charge [14], [18]. Second, subtypes of AF (paroxysmal, persistent or permanent) can not be classified because of the lack of specific ICD-9 codes. Third, personal information such as body mass index, glucose, lipid profiles, creatinine levels, alcohol use, and tobacco use are not available in the dataset. Fourth, the patients with hiatal hernia could not be identified in our database. Possible association between hiatal hernia and AF is an interesting question and worthy of future study, but it is outside the scope of the present paper. Finally, AF may be clinically silent and therefore underestimated. Our findings suggest that GERD may be associated with clinically significant AF. Studies designed to take clinically silent AF into account are still needed.

## Conclusions

The present study demonstrates an association between GERD and future development of AF, suggesting GERD may play a significant role in determining future risk of AF occurrence.
